# Nivolumab rechallenge after pituitary apoplexy associated with nivolumab plus ipilimumab in a patient with pituitary adenoma and rectal melanoma: a case report and literature review

**DOI:** 10.1186/s40780-026-00564-4

**Published:** 2026-03-19

**Authors:** Chiaki Imai, Masashi Uchida, Koji Takahashi, Izumi Ohno, Yuichi Takiguchi, Itsuko Ishii

**Affiliations:** 1 Division of Pharmacy, Chiba University Hospital, 1-8-1 Inohana, Chuo-ku, Chiba, Chiba 260-8677 Japan; 2 Department of Gastroenterology, Eastern Chiba Medical Center, 3-6-2 Okayamadai, Togane, Chiba 283-8686 Japan; 3 Department of Medical Oncology, Graduate School of Medicine, Chiba University, 1-8-1 Inohana, Chuo-ku, Chiba, Chiba 260-8670 Japan; 4 Department of Gastroenterology, Graduate School of Medicine, Chiba University, 1-8-1 Inohana, Chuo-ku, Chiba, Chiba 260-8670 Japan; 5 Department of Medical Oncology and Respiratory Medicine, Sannoh Hospital, 166-2 Sannoh-cho, Inage-ku, Chiba, Chiba 263-0002 Japan

**Keywords:** Pituitary apoplexy, Immune checkpoint inhibitors, Ipilimumab, Nivolumab, Adverse events, Melanoma

## Abstract

**Background:**

The overall incidence of pituitary apoplexy among patients with pituitary adenoma ranges from 0.6% to 7.0%, although many cases likely go undiagnosed. Several precipitating factors have been identified, and a small number of case reports have suggested an association between immune checkpoint inhibitors and pituitary apoplexy, particularly in patients with preexisting adenoma. Moreover, the safety of immune checkpoint inhibitor rechallenge remains uncertain.

**Case presentation:**

The patient was a 71-year-old woman with a recurrence of metastatic primary rectal melanoma. A pituitary tumor had been identified before initiation of systemic therapy. Following the second cycle of nivolumab plus ipilimumab, the patient developed headache, nausea, and vomiting, followed by decreased serum thyroid-stimulating hormone, adrenocorticotrophic hormone, and luteinizing hormone levels. These findings were consistent with evolving hypopituitarism. Contrast-enhanced magnetic resonance images revealed hemorrhage in the pituitary adenoma, suggesting pituitary apoplexy. Eight months after discontinuation of nivolumab plus ipilimumab, disease progression was noted, and nivolumab monotherapy was resumed. There was no recurrence of pituitary apoplexy during six cycles of nivolumab.

**Conclusions:**

This case highlights that pituitary apoplexy can occur temporally after nivolumab plus ipilimumab treatment in patients with preexisting pituitary adenoma. Importantly, nivolumab monotherapy rechallenge under close monitoring may be feasible without recurrence of pituitary apoplexy.

## Background

Pituitary apoplexy is a rare clinical condition, with an estimated incidence of 0.17 episodes per 100,000 person-years [1]. Pituitary adenoma has been reported to be associated with substantial morbidity and potential mortality. Among patients with preexisting pituitary adenoma, the incidence of pituitary apoplexy ranges from 0.6% to 7.0%, although the diagnosis may be missed in many cases [2]. The clinical manifestations of pituitary apoplexy are highly variable and depend largely on the extent of hemorrhage, necrosis, and associated edema. The hallmark symptom is sudden and severe headache, which is frequently accompanied by visual disturbance or ocular motor palsy [3]. Given its acute and often dramatic clinical presentation, pituitary apoplexy is considered to be an endocrine emergency that necessitates multidisciplinary management. Four mechanisms have been proposed for the development of pituitary apoplexy [4]: (1) reduction in vascular perfusion caused by, for example, surgery (particularly cardiac surgery), radiotherapy, or following spinal anesthesia; (2) an acute increase in blood flow, including physical exertion and systemic hypertension; (3) pituitary stimulation, as seen with provocative tests, particularly thyrotropin-releasing hormone and gonadotropin-releasing hormone analogs; and (4) coagulation disturbances, such as thrombocytopenia or anticoagulant therapy. Several risk factors for pituitary apoplexy have been identified, including pregnancy, diabetes mellitus, sickle cell anemia, estrogen replacement therapy, dopamine agonist therapy, lymphocytic leukemia, and head trauma [1]. The most frequent risk factor is hypertension, which is observed in 40% of patients with pituitary apoplexy [5], followed by anticoagulant therapy and direct oral anticoagulants [6]. Although rare, emerging evidence from case reports suggests a possible association between immune checkpoint inhibitor (ICI) therapy and pituitary apoplexy, especially in patients with preexisting adenoma [7–10]. ICIs remain a key treatment for advanced melanoma [11], and conventional cytotoxic chemotherapy has historically provided only limited benefit in mucosal melanoma [12]. Therefore, clarifying the feasibility and safety of ICI rechallenge after severe endocrine events is clinically important, but remains uncertain.

The present report describes a case of pituitary apoplexy associated with nivolumab plus ipilimumab in a patient with a preexisting pituitary adenoma and rectal (mucosal) melanoma, and highlights the potential feasibility of subsequent nivolumab monotherapy rechallenge without recurrence of pituitary apoplexy.

## Case presentation

**Fig. 1:**
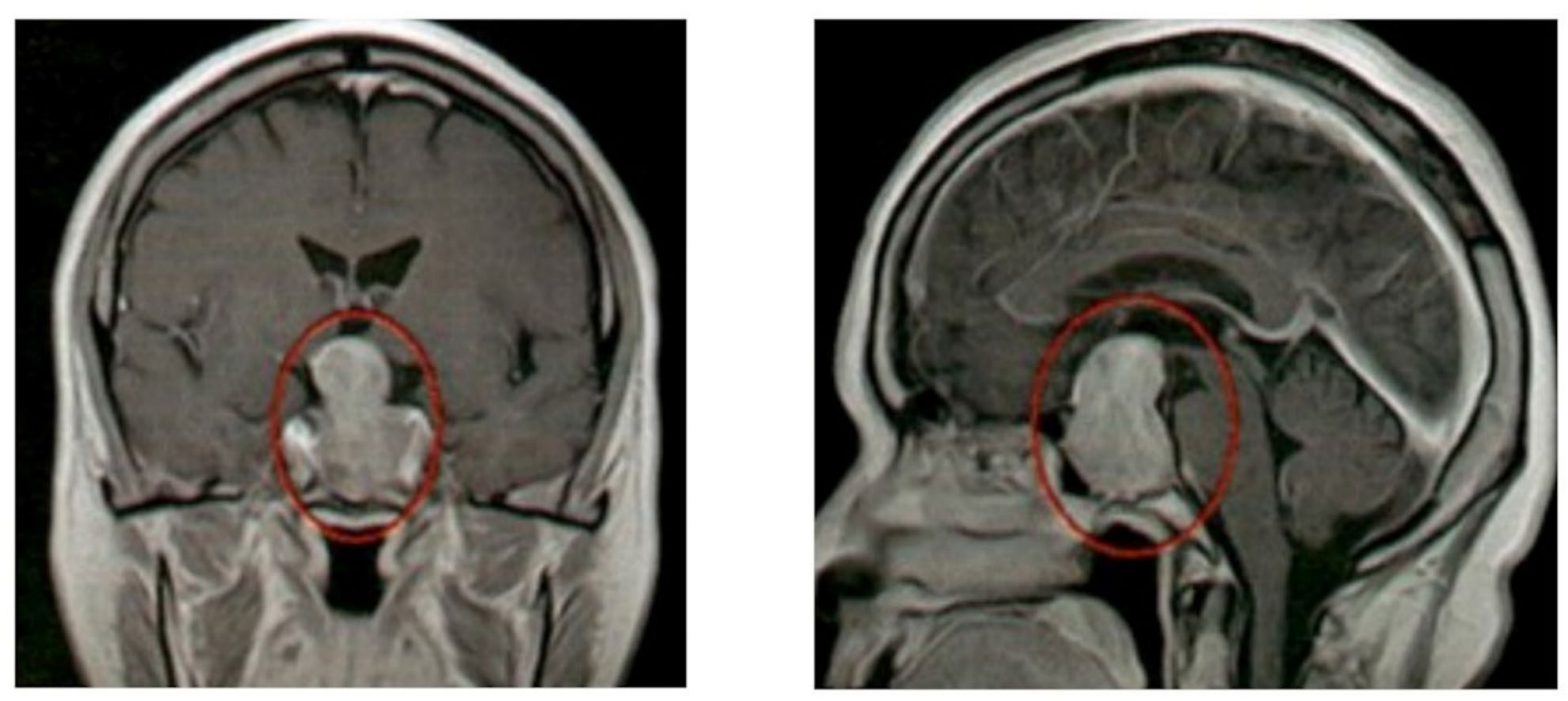
Magnetic resonance imaging before therapy. Magnetic resonance imaging of the brain showing a sellar and suprasellar mass requiring differential diagnosis of pituitary adenoma and clinically suspected to be pituitary metastasis from malignant melanoma. Red circles indicate the pituitary mass

A 71-year-old woman was referred to our hospital for further management of a recurrence of melanoma following resection of the primary rectal tumor. Two years earlier, she had presented to another hospital with bloody stool. Colonoscopy had revealed a black-pigmented rectal tumor, and subsequent endoscopic submucosal dissection confirmed a diagnosis of melanoma. She remained asymptomatic until 2 years later, when she experienced gross hematuria that was thought to be caused by recurrence of melanoma. Examinations performed at the previous hospital and our institution, including anal inspection, digital rectal examination, computed tomography (CT), and positron emission tomography, revealed scattered black-pigmented lesions at the anus, tumors in the rectum, and metastases to the hepatic, para-aortic, and pelvic lymph nodes as well as the liver, uterus, and lumbar vertebrae. A skin biopsy from an anal lesion confirmed recurrent melanoma, showing negative programmed death-ligand 1 (PD-L1) expression and wild-type B-Raf proto-oncogene, serine/threonine kinase (*BRAF)* on molecular testing. Magnetic resonance imaging (MRI) of the brain performed for staging revealed a mass extending from the sella turcica to the suprasellar region, necessitating differential diagnosis between pituitary metastasis and pituitary adenoma (Fig. 1). Laboratory findings before initiation of drug therapy revealed normal thyroid and adrenal function (Table 1). The patient’s concomitant medication at baseline included amlodipine 5 mg. Her blood pressure was well controlled throughout the course (approximately 120–130/70–80 mmHg). No anticoagulant or antiplatelet drugs were used.

**Table 1:** Changes in laboratory parameters after onset of pituitary apoplexy

Parameter	Unit of measurement	Reference range	(Prior to the cycle 1) Baseline^†^	(Prior to the cycle 2) Day 1^‡^	Day 4	Day 7	Day 9	One month	Two months
TSH	µIU/mL	(0.35–4.94)	1.93	0.72	**0.22**	–	**0.08**	**0.03**	**0.06**
FT3	pg/mL	1.68–3.67	–	–	1.91	–	**< 1.50**	1.98	2.72
FT4	ng/mL	0.70–1.48	0.79	0.82	0.97	–	0.76	0.95	1.21
ACTH	pg/mL	< 63.3	34.5	–	19.4	–	–	**< 1.50**	**< 1.50**
Cortisol	µg/dL	7.10–19.6	8.8	–	12.1	–	–	34.1	13.7
Eosinophil percentage	%	1.0–5.0	2.6	**11.0**	**11.0**	**21.0**	**9.9**	3.3	–
Serum sodium	mmol/L	138–145	141	140	**132**	**134**	**137**	142	142
CRP	mg/dL	0.00–0.14	0.14	**0.21**	**0.2**	**0.65**	**0.46**	0.11	0.14
FSH	mIU/mL	2.58–151	–	–	7.06	–	–	–	8.11
LH	mIU/mL	5.7–64.3	–	–	**0.6**	–	–	–	**0.7**
PRL	ng/mL	5.18–26.53	–	–	21.8	–	–	–	**4.2**
Estradiol	pg/mL	< 10–28	–	–	< 10	–	–	–	< 10
Progesterone	ng/mL	< 0.1–0.2	–	–	< 0.1	–	–	–	< 0.1
GH	ng/mL	0.13–9.88	–	–	0.85	–	–	–	0.23

Baseline^†^, Laboratory values obtained before administration of the first course of nivolumab plus ipilimumab. Day 1^‡^, Laboratory values obtained before administration of the second course of nivolumab plus ipilimumab, pituitary apoplexy developed on the night of the same day after infusion of immune checkpoint inhibitor therapy. The values shown in bold are outside the reference range. Abbreviations: –, not tested; <, below detection limit. ACTH, adrenocorticotrophic hormone; CRP, C-reactive protein; FSH, follicle-stimulating hormone; FT3, free triiodothyronine; FT4, free thyroxine; GH, growth hormone; LH, luteinizing hormone; PRL, prolactin; TSH, thyroid-stimulating hormone

Combination therapy with nivolumab (80 mg every 3 weeks) + ipilimumab (3 mg/kg every 3 weeks) was initiated. The cause of the gross hematuria was unclear; however, urinalysis after the first course of melanoma treatment did not detect hematuria.

**Fig. 2:**
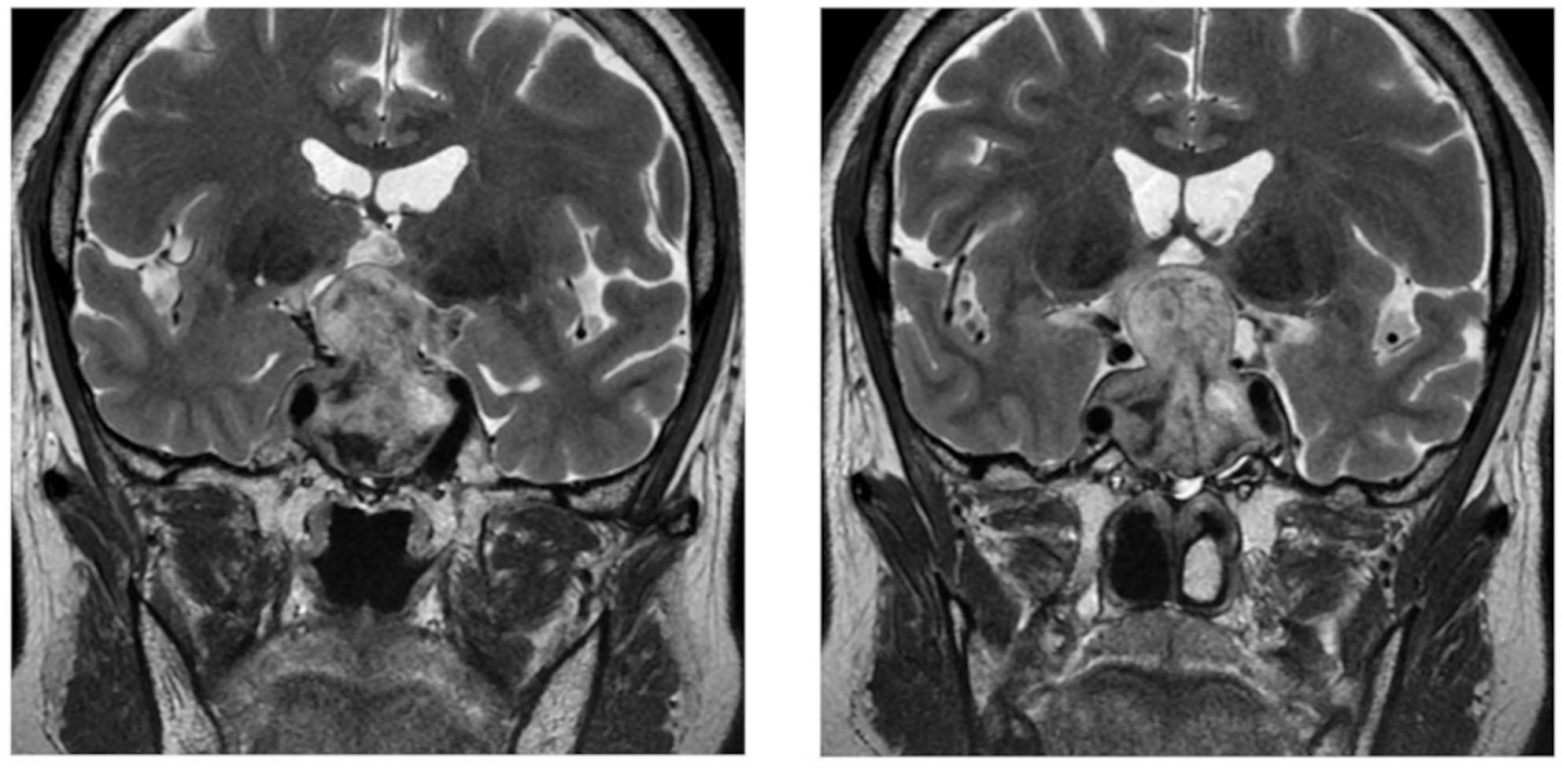
Contrast-enhanced magnetic resonance imaging on day 8 after onset of pituitary apoplexy. Contrast-enhanced magnetic resonance images showing a 33 × 27 × 43 mm mass extending from the intrasellar to suprasellar region. The lesion contained heterogeneous high-signal intensity on T2-weighted images, suggesting hemorrhage and necrosis

Table 2 summarizes the clinical course and interventions. The patient developed a severe headache on the evening of the second-cycle infusion, followed 3 days later by chills, nausea, and vomiting. Worsening nausea, vomiting, and headache, despite repeated administrations of loxoprofen sodium (60 mg as needed) and acetaminophen (2,000 mg/day in four divided doses), prompted urgent evaluation. Neurological examination revealed bitemporal hemianopia, suggesting progression of the pituitary lesion. Although a brain CT scan performed at that time did not reveal any findings beyond the known pituitary lesion, contrast-enhanced MRI demonstrated an intrasellar-to-suprasellar mass with heterogeneous high signal intensity on T2-weighted images, suggesting hemorrhage and necrosis within the pituitary adenoma (Fig. 2). Subarachnoid hemorrhage and bacterial meningitis were considered; however, the patient was afebrile and had no neck stiffness or altered mental status, and non-contrast head CT showed no evidence of subarachnoid hemorrhage. In addition, bitemporal hemianopia supported a sellar/suprasellar process, and MRI findings were consistent with intratumoral hemorrhage within the pituitary adenoma rather than subarachnoid bleeding. Serial endocrine evaluations (Table 1) demonstrated progressive declines in serum thyroid-stimulating hormone (TSH) and adrenocorticotrophic hormone (ACTH), further implicating a lesion within the pituitary as the cause. The baseline luteinizing hormone (LH) level was unavailable; however, the post-apoplexy LH level was confirmed to be below the reference range. Interestingly, the peripheral eosinophil count increased markedly from baseline on day 1 of the second cycle, rising from 2.6% (182/µL) to 11.0% (924/µL). Based on the above findings, a pituitary apoplexy was diagnosed. Thereafter, the patient’s symptoms waxed and waned, with recurrent ptosis accompanying persistent headache despite treatment with hydrocortisone (50 mg/day), non-steroidal anti-inflammatory drugs, and acetaminophen. Ultimately, maintaining hydrocortisone at 100 mg daily for 5 consecutive days resulted in resolution of all symptoms. Levothyroxine was initiated at 25 µg/day on day 9 of the second cycle and continued thereafter; free triiodothyronine (FT3) and free thyroxine (FT4) remained within the reference ranges at 1 and 2 months after the onset of pituitary apoplexy (Table 1). Hydrocortisone was tapered to 30 mg/day orally (20 mg morning, 10 mg evening) without recurrence of symptoms, and the melanoma remained stable for 7 months without additional anticancer therapy. However, a planned restaging CT revealed significant enlargement of the para-aortic lymph node metastases. Nivolumab was resumed as monotherapy because therapeutic alternatives were limited and disease progression required systemic treatment. Given the previous event during combination therapy, we selected nivolumab monotherapy rather than reintroducing ipilimumab. Prior to rechallenge, the treating physician obtained informed consent after discussing the expected benefits of nivolumab monotherapy (tumor control, symptom relief, and potential survival benefit), the potential risk of recurrent pituitary apoplexy and other serious immune-related adverse events (irAE), alternative options including dacarbazine (with expected limited efficacy) and best supportive care, and the possibility that the disease could progress over months and become life-threatening (approximately within 3–6 months) without effective systemic therapy. During pharmacist counseling, the patient expressed anxiety about adverse events but wished to continue treatment in the hope of clinical benefit. After six cycles, contrast-enhanced CT demonstrated stable disease. At that point, the patient reported blurred vision and was diagnosed with bilateral uveitis, which was considered to be an irAE. Following discontinuation of nivolumab, the visual disturbances improved spontaneously without any additional therapy. Considering that the patient’s condition remained stable, an endoscopic endonasal transsphenoidal resection of the pituitary tumor was performed. Histopathological examination confirmed a pituitary adenoma with negative immunohistochemical staining for ACTH, TSH, and LH, which was consistent with the clinical presentation.

**Table 2:** Clinical timeline

Time point	Key events / clinical findings	Management / interventions
20XX June	Rectal melanoma diagnosed and resected	Underwent transanal proctocolectomy
Two years later, in March to November	Recurrence with metastatic disease confirmed (liver/lymph node/uterus/lumbar vertebra). At referral, PD-L1 negative and BRAF wild-type; brain MRI showed a sellar–suprasellar mass (adenoma vs. metastasis).	Staging and treatment planning; pituitary lesion monitored
Next year February (Cycle 1 Day 1)	Nivolumab plus ipilimumab initiated	Nivolumab plus ipilimumab (q3w)
**Cycle 2 Day 1** ^**‡**^	Headache began in the evening after the second infusion	None
Days 3–6	Headache ± chills/nausea/vomiting; refractory to oral medications	Loxoprofen/acetaminophen → admitted on Day 4 for symptom control; suspected pituitary apoplexy vs. ICI-associated hypophysitis; IV HC 100 mg (transient relief); hydroxyzine/pentazocine as needed
Days 7–8	Persistent headache/nausea; suspected secondary adrenal insufficiency	IV HC 50 mg re-start → improved; CE-MRI consistent with hemorrhagic/degenerative pituitary apoplexy
Days 9–10	Right ptosis; visual disturbance; recurrence of headache/nausea	HC 50 mg continued; started LT4 25 µg (TSH 0.08 µIU/mL, FT4 0.76 ng/mL); extra HC 100 mg (day 10)
Days 11–14	Ptosis/visual symptoms/headache/nausea improved	IV HC 100 mg/day q24h ×4 days; LT4 continued
Days 15–17	Asymptomatic	Tapered to oral HC 30 mg/day; LT4 25 µg/day continued
Day 18	Asymptomatic	Discharge on oral HC 30 mg/day + LT4 25 µg/day
~ 8 months later	Disease progression after discontinuation of nivolumab plus ipilimumab	Decision-making for systemic therapy
8 ~ 13 months later	Nivolumab monotherapy resumed	No recurrence of pituitary apoplexy during subsequent cycles; discontinued due to uveitis (irAE), which improved spontaneously

Day 1^**‡**^, The day of the second course of nivolumab plus ipilimumab administration.  Abbreviations: BRAF, B-Raf proto-oncogene, serine/threonine kinase; CE-MRI, contrast-enhanced magnetic resonance imaging; FT4, free thyroxine; HC, hydrocortisone; ICI, immune checkpoint inhibitor; irAE, immune-related adverse event IV; intravenous injection; LT4, levothyroxine; MRI, magnetic resonance imaging; PD-L1, programmed death-ligand 1; TSH, thyroid-stimulating hormone

## Discussion and conclusions

In this patient, pretreatment staging for recurrent rectal melanoma revealed a pituitary tumor. A definitive differential diagnosis between pituitary adenoma and pituitary metastasis from melanoma had not been established, and combination immunotherapy with nivolumab plus ipilimumab was initiated. At the time of evaluation for acute onset of headache, nausea, and vomiting, the pituitary lesion remained stable in size, whereas other metastatic lesions decreased after two cycles of combination therapy. This finding reduced the likelihood that the pituitary tumor was a metastasis. In view of the decreased serum TSH, ACTH, and LH levels, the differential diagnosis at that point included pituitary apoplexy, pseudoprogression of pituitary metastasis, and ICI-induced hypophysitis. Close review of contrast-enhanced MRI scans led to the conclusion that the findings represented pituitary apoplexy within a preexisting pituitary adenoma. Finally, histopathological examinations of the resected pituitary tumor, including immunohistochemistry, confirmed a pituitary adenoma. The absence of immunohistochemical staining for adenohypophyseal (anterior pituitary) hormones was consistent with pituitary apoplexy. In the present case, endocrine abnormalities did not occur immediately after treatment initiation; TSH first became clearly abnormal on day 25 of the first cycle (0.22 µIU/mL) (Table 1). Although temporal relationship and clinical findings support a possible association between pituitary apoplexy and combined ICI therapy, other contributing factors cannot be fully excluded.

The next question concerns the mechanisms underlying pituitary apoplexy associated with combined ICI therapy. ICIs, particularly ipilimumab, an IgG1 subclass anti-cytotoxic T-lymphocyte-associated protein 4 (CTLA-4) antibody, cause hypophysitis, with reported incidences ranging from 1.8% to 17.0% [13]. This is plausibly because normal pituitary glands contain cells that express CTLA-4, and CTLA-4 blockade can lead to complement-mediated injury [14]. Hypophysitis may promote further enlargement of a pre-existing pituitary adenoma within the confined space of the sella turcica, leading to mass effect and altered vascular perfusion, both of which are pathophysiological changes implicated in pituitary apoplexy [3, 15–17]. In anti-CTLA-4 antibody-induced hypophysitis, MRI abnormalities, such as pituitary enlargement or stalk thickening, are reported in approximately 81% of cases. In contrast, MRI abnormalities are observed in only about 18% of patients with anti- Programmed death 1 (PD-1) or PD-L1-induced hypophysitis [15, 18]. These changes may precipitate intratumoral hemorrhage and necrosis within the pituitary adenoma, culminating in pituitary apoplexy. This hypothesis appears to be consistent with the clinical course in this case; that is, combination therapy with nivolumab plus ipilimumab was associated with the onset of pituitary apoplexy, whereas subsequent nivolumab monotherapy did not result in recurrence. Elevated peripheral eosinophil counts after combination therapy may support the presence of an irAE [19–21]. Although definitive evidence supporting this hypothesis was lacking in this patient, it is theoretically plausible that hypophysitis as an irAE arising in a preexisting pituitary adenoma could precipitate pituitary apoplexy.

A literature search identified four case reports of pituitary apoplexy occurring during ICI therapy (Table 3) [7–10]. Similar to our patient, these cases were reported in the setting of a preexisting pituitary mass, suggesting that an underlying adenoma may be a common background factor. One of the four cases was received a combination of nivolumab and ipilimumab [9]. Notably, the patient in the report was subsequently re-treated with nivolumab monotherapy after discontinuation of the combination regimen. However, detailed outcomes regarding recurrence of pituitary apoplexy were not described [9]. Our case provided clinically relevant practice of rechallenging nivolumab monotherapy without recurrence of pituitary apoplexy over six cycles under careful monitoring. Hypophysitis has been reported in up to 10% of patients receiving anti-CTLA-4-based therapy, including ipilimumab monotherapy [22]. In contrast, with anti-PD-1 or anti-PD-L1 monotherapy, hypophysitis occurs in only 0.5%–1.0% of patients and is considered rare compared with CTLA-4 blockade [22]. Moreover, in the treatment of various malignancies worldwide, anti-PD-1/PD-L1 antibodies are administered far more frequently than anti-CTLA-4 antibodies. This may explain the apparent discrepancy whereby our literature search identified more cases of pituitary apoplexy associated with anti-PD-1/PD-L1 therapy than with anti-CTLA-4 therapy, despite the higher reported incidence of hypophysitis with CTLA-4 blockade. Peripheral eosinophilia preceded overt symptoms in our case, whereas eosinophilia was not documented in the previous case reports. This observation may have practical implications for monitoring: an unexpected rise in eosinophil count during ICI therapy, particularly with ipilimumab-containing regimens, may serve as an early clue to immune-mediated endocrinopathy and prompt earlier evaluation and intervention.

Practical implications for early detection: Because pituitary apoplexy may be missed, patients with a preexisting pituitary adenoma receiving ipilimumab-containing ICI therapy should be monitored closely for red-flag symptoms (sudden severe headache, nausea/vomiting, and visual disturbance). In our case, peripheral eosinophilia preceded overt symptoms and may serve as a potential early clue to immune-mediated endocrinopathy. Pharmacists can support early intervention by proactively sharing these risks with the multidisciplinary team, educating patients, and prompting endocrine evaluation (e.g., morning cortisol/ACTH, electrolytes, thyroid function) and pituitary imaging when eosinophils increase or symptoms of pituitary apoplexy appear. Such coordination may facilitate earlier diagnosis and treatment.

In conclusion, in patients with a preexisting pituitary adenoma who developed pituitary apoplexy associated with nivolumab plus ipilimumab therapy, nivolumab monotherapy rechallenge may be feasible under careful multidisciplinary monitoring.

**Table 3:** Reported cases of pituitary apoplexy during immune checkpoint inhibitor therapy

Authors (year)	Cancer/ICI	Clinical context and timing	Snapshot (symptoms, laboratory results, imaging, treatment, ICI continuation)
Carril-Ajuria et al. (2018) [7]	RCC/anti-PD-1	Unrecognized macroadenoma; on ICI	Headache, nausea, asthenia; ↓TSH, ↓FT3, normal FT4, ↓testosterone; hemorrhagic apoplexy on MRI; treatment with high-dose steroids + LT4; ICI, not reported
Lopes et al. (2023) [8]	MM/pembrolizumab	Known macroadenoma; +2 months	Severe headache; primary, central hypogonadism; mild ↑PRL; later, new central hypothyroidism + adrenal insufficiency (persisting for 3 months); apoplexy on MRI; treatment with CS and LT4→ surgery. ICI, not reported
Uddin et al. (2023) [9]	MM/nivolumab and ipilimumab	Presumed prolactinoma; after Cycle 4	Headache, confusion; pre, ↑PRL, ↑FSH/LH with normal testosterone, borderline ↑TSH with normal FT4; post, hyponatremia, central hypogonadism, hypothyroidism, adrenal insufficiency. enlargement on MRI; hypophysitis suspected. Treated with HRT + correction of hyponatremia. ICI, continued (nivolumab)
Hayhurst et al. (2025) [10]	NSCLC/atezolizumab	Known macroadenoma; after Cycle 2	Headache, fever, confusion; ↓IGF-1; central hypothyroidism and hypogonadism; normal PRL. infarction + hemorrhage on MRI. Treated with high-dose steroids + lifelong HRT. ICI stopped

↑/↓, above/below reference range; CS, corticosteroids; FSH, follicle-stimulating hormone; FT3, free triiodothyronine; FT4, free thyroxine; HRT, hormone replacement therapy; ICI, immune checkpoint inhibitor; IGF-1, insulin-like growth factor 1; LH, luteinizing hormone; LT4, levothyroxine; MM, malignant melanoma; MRI, magnetic resonance imaging; NSCLC, non-small cell lung cancer; PD-1, programmed death 1; PRL, prolactin; RCC, renal cell carcinoma; TSH, thyroid-stimulating hormone

## Data Availability

All data generated or analyzed during this study are included in this published article.

## Abbreviations

ACTH: Adrenocorticotrophic hormone
BRAF: B-Raf proto-oncogene, serine/threonine kinase
CE-MRI: Contrast-enhanced magnetic resonance imaging
CRP: C-reactive protein
CS: Corticosteroid
CT: Computed tomography
CTLA-4: Cytotoxic T-lymphocyte-associated protein 4
FSH: Follicle-stimulating hormone
FT3: Free triiodothyronine
FT4: Free thyroxine
GH: Growth hormone
HC: Hydrocortisone
HRT: Hormone replacement therapy
ICI: Immune checkpoint inhibitor
IGF-1: Insulin-like growth factor 1
irAE: Immune-related adverse event
IV: Intravenous
LH: Luteinizing hormone
LT4: Levothyroxine
MM: Malignant melanoma
MRI: Magnetic resonance imaging
NSCLC: Non-small cell lung cancer
PD-1: Programmed death 1
PD-L1: Programmed death-ligand 1
PRL: Prolactin
RCC: Renal cell carcinoma
TSH: Thyroid-stimulating hormone
